# Study of the Dielectric Properties of Artificial Sweat Mixtures at Microwave Frequencies

**DOI:** 10.3390/bios10060062

**Published:** 2020-06-09

**Authors:** Angie R. Eldamak, Sarah Thorson, Elise C. Fear

**Affiliations:** 1 Electronics and Electrical Communication Department, Faculty of Engineering, Ain Shams University, Cairo 11517, Egypt; 2 Schulich School of Engineering, University of Calgary, Calgary, AB T2N1N4, Canada; sarahgracethorson@gmail.com (S.T.); fear@ucalgary.ca (E.C.F.)

**Keywords:** dielectric spectroscopy, dielectric properties, artificial sweat, sweat electrolytes, sweat monitoring, hydration monitoring

## Abstract

Analysis of sweat is of interest for a variety of diagnosis and monitoring applications in healthcare. In this work, detailed measurements of the dielectric properties of solutions representing the major components of sweat are presented. The measurements include aqueous solutions of sodium chloride (NaCl), potassium chloride (KCl), urea, and lactic acid, as well as their mixtures. Moreover, mixtures of NaCl, KCl, urea, and lactic acid, mimicking artificial sweat at different hydration states, are characterized, and the data are fitted to a Cole–Cole model. The complex dielectric permittivity for all prepared solutions and mixtures is studied in the range of 1–20 GHz, at temperature of 23 °C, with ionic concentrations in the range of 0.01–1.7 mol/L.

## 1. Introduction

With the increasing market in wearable devices, health monitoring, and preventive medicine, there is growing interest in analyzing properties of biofluids in the microwave frequency range. Dielectric properties of these fluids contribute to the understanding of microwave interaction with biological tissues [1,2] and allow identification of opportunities for disease detection and prevention [3,4,5]. This knowledge also contributes towards testing the impact of biofluids, specifically sweat, on health monitoring devices [5,6] and consumer products [7,8]. Thus, accurate knowledge of dielectric properties of biofluids, specifically sweat, supports the design of new sensors, wearable devices, and therapeutic technologies.

Biofluids, including urine, blood, tears, and sweat, carry physiological biomarkers that can reflect health status [5,6,9]. Among the different biofluids, the composition of sweat and blood are osmotically related Tricoli et al. [5]. Although blood carries highly accurate information on the human body, sweat has the potential for easy, fast, and noninvasive monitoring Tricoli et al. [5].

Human sweat is composed of metabolites (uric acid, urea, and lactic acid), minerals (sodium, chloride, potassium, magnesium, zinc, iron, calcium, copper, and phosphate), as well as amino acids [3,5,9,10,11,12,13,14]. Among these components, tracking sodium (Na^+^), chloride (Cl^−^), and potassium (K^+^) can provide information on the water–salt balance in human tissues Romanov [4], hydration levels [11,12,13,14,15,16,17,18,19,20], and the presence of cystic fibrosis [3,5,21]. Properties of sweat in the microwave frequency range were reported in Romanov [4], which focused on comparing dielectric properties of sweat collected from different locations on the body. The work presented in Romanov [4] used an industrial phasometer to measure transmission magnitude and phase, which were used to calculate dielectric properties of sweat in the range of 300 MHz to 3 GHz.

Artificial sweat mixtures have been developed to mimic eccrine perspiration and used commercially to test consumer products such as bank cards, textiles, jewelry, and leather [7,8]. Other studies have used artificial sweat mixtures as test liquids when developing sensors [11,12,13]. Several technical associations have released different standards describing artificial sweat formulations [7,8,10,11,12,13]. From [7,8,10,11,12,13], the major components of artificial sweat are sodium chloride (NaCl), potassium chloride (KCl), urea, and lactic acid. 

Several studies characterized the frequency-dependent properties of solutions of NaCl and KCl at different temperatures and concentrations from 100 kHz to 40 GHz in [1,2,3,11,22,23]. In Gulich et al. [1], Peyman et al. [2] and Nörtemann et al. [3], dielectric properties of NaCl and KCl solutions over the temperature range of 10–60 °C and concentration range of 0.001–5 mol/L were presented in the band from 100 MHz to 40 GHz. Moreover, the measurements in Peyman et al. [2] and Nörtemann et al. [3] were combined with literature values to derive empirical equations to describe the dielectric behavior of NaCl solutions using Debye and Cole–Cole models. In Lamkaouchi et al. [22], the permittivity of aqueous solutions of NaCl at millimeter wave bands of 37, 89, and 110 GHz over the temperature range of 0–25 °C was also reported. The effect of temperature, pressure, and salt concentrations (up to 6 mol/L) on the permittivity of several aqueous salt solutions was examined in Maribo-Mogensen et al. [23]. The tested NaCl concentrations in Maribo-Mogensen et al. [23] are as high as 35% and do not represent concentrations in biofluids. However, all data in Maribo-Mogensen et al. [23] refer to static permittivity and the effect of varying frequency was not studied. None of the reported studies examined the properties of mixtures or properties in the presence of other components of sweat, namely, urea and lactic acid. In Liu et al. [11], the conductivity of different concentrations of artificial sweat (14–262 mmol) was measured at different temperatures and at the single frequency of 100 kHz. The properties of urea in saline solutions were studied in the 1–14 GHz band in Jensen et al. [24] as a biomarker for dialysis treatment and kidney dysfunction. Electrical properties of lactate were reported in De los Reyes et al. [25] for agriculture applications.

Artificial sweat mixtures representing different hydration states were synthesized by our group in Eldamak et al. [26] to test an antenna-based sensor for noninvasive sweat monitoring. Electrical properties for artificial sweat mixtures representing normal and dehydrated sweat were reported in Eldamak et al. [26]. Although varying NaCl concentrations were tested, the impact of varying concentrations of other components of the artificial sweat solution was not explored in detail. This study presents the dielectric properties of aqueous solutions of the significant electrolytes in sweat, mixtures of these components, and artificial sweat mixtures representing normal and dehydration states. The dielectric properties are measured over the range of 1–20 GHz. This paper also explores the effect of pH level on electrical properties and provides Cole–Cole parameters for artificial sweat (normal and dehydrated concentrations) to fit measured data.

## 2. Materials and Methods

### 2.1. Materials and Tested Solutions

The preparations of artificial sweat (AS) follow the EN1811:2011 European Standard [8,10,11,12,13]. The initial recipe in Midander et al. [8] and Callewaert et al. [10] mimics human perspiration to test nickel release from jewelry as sweat can react with certain materials and trigger dermatitis nickel allergy or shorten product service life [7]. This recipe included 0.5% NaCl, 0.1% urea, and 0.1% lactic acid dissolved in 1 L of distilled water. However, this recipe was modified in Liu et al. [11], Liu et al. [12] and Hoekstra et al. [13] to test sweat monitoring devices. Specifically, 0.1% potassium chloride (KCl) was added to match the composition of human sweat reported in Liu et al. [12], Baker et al. [14] and Morgan et al. [15]. The modified recipe used in Liu et al. [11], Liu et al. [12] and Hoekstra et al. [13] was adopted in this paper.

The solution representing dehydrated sweat involved dissolving 85 mmol of sodium chloride (NaCl), 13 mmol of potassium chloride (KCl, Fisher Scientific), 17 mmol of lactic acid (LD CARLSON Co., food-grade Lactic Acid 88%), and 16 mmol of urea (Jacquard, Commercial Grade) in 1 L of distilled water. For normal artificial sweat, the proportions of components were kept in the same ratios but the concentrations were one-tenth of those representing dehydration [11,24]. In addition, the pH level for all tested solutions was recorded using pH indicator paper with range of 0–14. All concentrations were in mol/L. In addition to the artificial sweat mixtures, test solutions with different concentrations of NaCl, KCl, lactic acid, and urea and their mixtures were investigated. The single component solutions and mixtures explored in this study are presented in Table 1 and Table 2, respectively. Table 1 summarizes single component solutions synthesized by dissolving the given amount of the tested component in 1 L of distilled water. Table 2 summarizes mixtures under test created by dissolving the given amounts of base and tested components in 1 L of distilled water. Properties of analyzed solutions were compared to distilled water. The focus was on concentrations in the range of 0.01–0.2 mol, which is a common range among all tested components.

### 2.2. Measurement Setup

**Figure 1:**
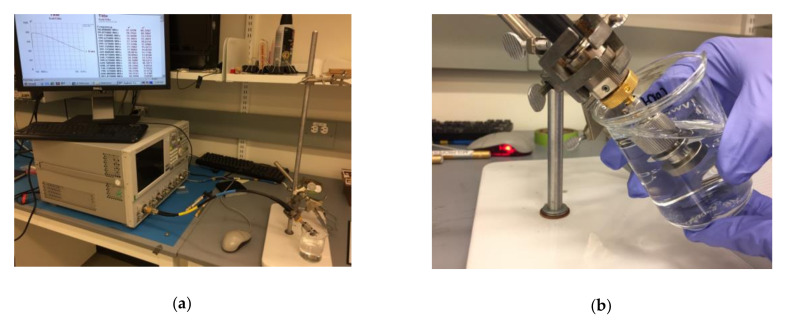
(**a**) Overall measurement setup including dielectric probe (87050E, Keysight Technologies), vector network analyzer (E8364B, Keysight Technologies), and solution under test (SUT) and (**b**) dielectric probe immersed in SUT.

All dielectric properties were measured using a dielectric probe and vector network analyzer (Agilent 87050E and E8364B, respectively), as well as the associated software as shown in Figure 1a. This technique is based on measuring reflection from an open-end coaxial probe immersed in the solution under test (SUT) [1,27]. Standard techniques were used to calibrate the measurement system and estimate complex permittivity from the reflection coefficient La Gioia et al. [27].

This frequency-dependent permittivity (ε(ω)*) was estimated in the range of 1–20 GHz at temperature of 23 °C. The frequency-dependent conductivity (σ(ω)) estimates, representing losses in the solutions under test, were obtained from the complex permittivity using Equation (1):

(1) $$\varepsilon {\left(\omega \right)}^{*}=\;\varepsilon \;\prime \left(\omega \right)-j\;\varepsilon \;\prime \prime \left(\omega \right)=\;\varepsilon \;\prime \left(\omega \right)-j\frac{\sigma \left(\omega \right)}{{\omega \varepsilon }_{o}}$$

where ω is the angular frequency, the real and imaginary parts of the complex permittivity are denoted by ε’ and ε”, respectively, and ε_o_ is the permittivity of vacuum. A 120 mL of sample of each prepared solution was measured using the set up shown in Figure 1. For each solution, three rounds of measurements were performed to minimize errors and confirm consistency. Each round involved collecting measurements at 400 equally spaced points from 1 to 20 GHz. The properties of each solution were estimated from the average of these three measurements. Each round involved also measuring properties of distilled water (DW) as a reference liquid.

### 2.3. Validation of Measurements Using NaCl Aqueous Solutions

**Figure 2:**
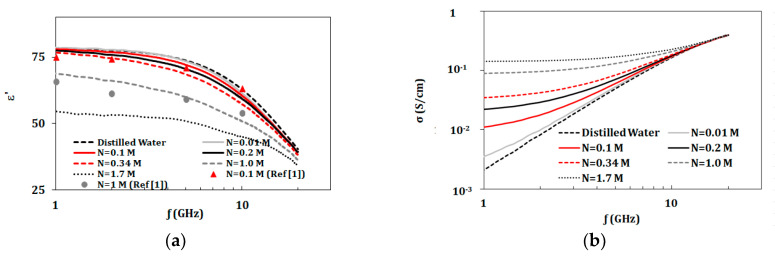
(**a**) Measured dielectric constant and (**b**) Measured conductivity for NaCl solutions at ion concentrations from 0.01 to 1.7 mol/L at temperature 23 °C in the range of 1–20 GHz (“N” refers to NaCl and “M” refers to mol/L).

To validate the measurements, solutions of NaCl with ion concentrations of 0.01–1.7 mol/L were characterized in the range of 1 to 20 GHz. The recorded data at different concentrations were compared to published data in Gulich et al. [1] as shown in Figure 2. Our measurements for solutions of NaCl showed an increase of 3–5% calculated over the band from 1 to 10 GHz when compared to measured and fitted data at similar concentrations, temperature, and frequency range reported in Gulich et al. [1].

## 3. Experimental Results

### 3.1. Single Component Solutions (KCl, Urea, and Lactic Acid)

**Figure 3:**
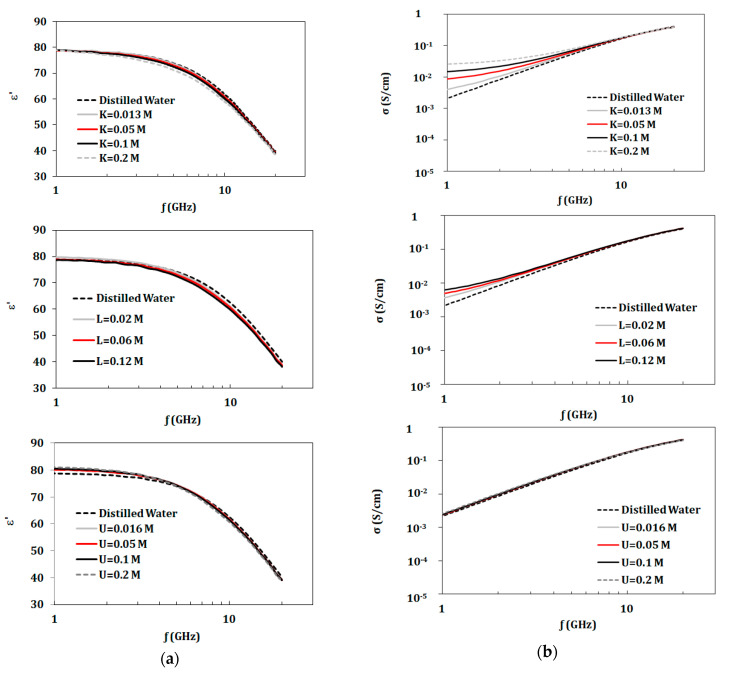
Measured dielectric properties for KCl, lactic acid, and urea solutions at ion concentrations 0.01 to 0.2 mol/L at temperature 23 °C in the range of 1–20 GHz: (**a**) dielectric constant and (**b**) conductivity. In all figures in this paper, “K” refers to KCl, “L” to lactic acid, “U” to urea, and “M” refers to mol/L.

In this section, dielectric properties of solutions of KCl, lactic acid, and urea (with concentrations given in Table 1) were investigated. Figure 2 and Figure 3 present dielectric properties for NaCl, KCl, lactic acid, and urea compared to distilled water in the band of 1–20 GHz. Properties of NaCl solutions were recorded for the larger concentration range from 0.01 to 1.7 mol. Other components were tested with concentrations up to 0.2 mol due to safety limitations. However, these solutions had concentrations of biological relevance.

### 3.2. Dual Component Solutions (NaCl and KCl)

For the given measurements shown in Figure 1 and Figure 2, NaCl and KCl had greatest impact on complex permittivity (ε’ and ε”) and conductivity values. Thus, it is interesting to study mixtures of these two components. In this section, two groups of measurements were presented. 

**Figure 4:**
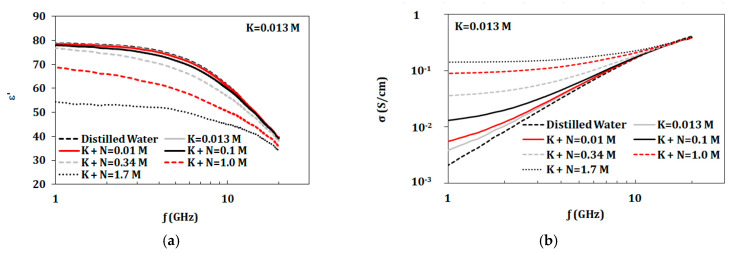
Measured dielectric properties for NaCl solutions at ion concentrations from 0.01 to 1.7 mol/L and KCl with fixed concentration of 0.013 mol/L at temperature 23 °C in the range of 1–20 GHz: (**a**) dielectric constant and (**b**) conductivity (“M” refers to mol/L).

**Figure 5:**
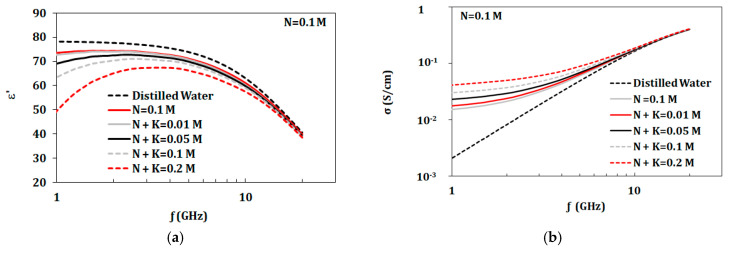
Measured dielectric properties for KCl solutions at ion concentrations from 0.01 to 0.2 mol/L and NaCl with fixed concentration of 0.1 mol/L at temperature 23 °C in the range of 1–20 GHz: (**a**) dielectric constant and (**b**) conductivity (“M” refers to mol/L).

The first group starts with a KCL concentration of 0.013 mol (corresponding to dehydrated artificial sweat) to 1 L of distilled water. NaCl was added to this given solution in concentrations in the range of 0.01–1.7 mol/L as shown in Table 2. The second group had fixed NaCl concentration of 0.1 mol (corresponding to dehydrated artificial sweat), whereas KCl was added in the range of 0.01–0.2 mol/L as shown in Table 2. Properties of both groups are presented in Figure 4 and Figure 5. From measurements shown in Figure 4a, with a base of 0.013 mol/L of KCl, the dielectric constant of mixtures did not showed significant change compared to distilled water until the NaCl concentration of 0.1 mol/L. Dielectric constant of solutions with a base of 0.1 mol/L of NaCl showed changes starting at KCl concentrations of 0.05 mol/L (Figure 5a). On the other hand, conductivity values were altered by adding either KCl or NaCl with concentrations as low as 0.01 mol/L as shown in Figure 4b and Figure 5b.

### 3.3. Artificial Sweat Mixtures

#### 3.3.1. Artificial Sweat Dielectric Properties

In this section, dielectric properties of artificial sweat solutions were investigated. Artificial sweat samples with concentrations described in Section 2.1 were synthesized. Two concentrations were considered, representing sweat concentration for normal (termed diluted) and dehydrated individuals.

**Figure 6:**
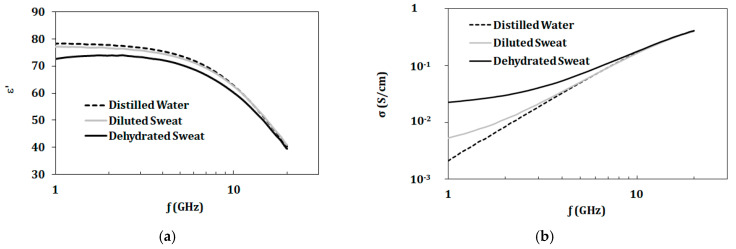
(**a**) Dielectric constant and (**b**) conductivity for distilled water (DW), diluted sweat (0.05% NaCl, 0.01% for each urea, lactic acid, and KCl), and dehydrated sweat (0.5% NaCl, 0.1% for each urea, lactic acid, and KCl.

Table 3 shows values of complex permittivity (ε* = ε’-j ε”) and conductivity for artificial sweat mixtures and distilled water at 2.45 GHz. From measurements shown in Figure 6, the dielectric constant of diluted sweat with 0.01 mol/L of NaCl was similar to distilled water in the 1–20 GHz band. However, diluted sweat exhibited greater conductivity in the lower frequencies of the band compared to distilled water. On the other hand, dehydrated sweat with NaCl concentrations of 0.1 mol/L showed distinct values for both dielectric constant and conductivity over the whole band. Moreover, the measured dielectric properties of the prepared artificial sweat mixtures showed similarity in values with those recorded for real sweat in Romanov [4]. 

#### 3.3.2. Effect of pH

The proposed artificial sweat mixtures had measured pH level of 4 after adding the lactic acid. The pH level of this solution was not in the rated range for human sweat. Ammonia and sodium hydroxide were mentioned in the recipes reported in Midander et al. [8], Callewaert et al. [10] and Liu et al. [12] as a means of adjusting pH level of artificial sweat from 4 to 6.5. These additives are components of artificial sweat from a physiological point of view. In this work, pH was adjusted to 6.5 using sodium bicarbonate instead of ammonia or sodium hydroxide due to safety limitations. The dielectric properties of dehydrated sweat mixtures with pH level of 4 and 6.5 were recorded. Measurements showed maximum variations of 3% in dielectric properties for solutions with pH level 4 or 6.5.

#### 3.3.3. Cole–Cole Model for Artificial Sweat Mixtures

In this section, the measured frequency-dependent dielectric properties for artificial sweat mixtures representing normal and dehydrated states were fitted to a single pole Cole–Cole model as in Gulich et al. [1], Peyman et al. [2] and Nörtemann et al. [3]. The model is the sum of a relaxation function and a contribution from conductivity as presented in Equation (2) as:

(2) $$\hat{\varepsilon }=\varepsilon_{\infty }+\frac{\varepsilon_{s}-\varepsilon_{\infty }}{1+{\left(j\omega \tau \right)}^{1-\alpha }}+\frac{\sigma_{i}}{{j\omega \varepsilon }_{o}},$$

where, ε_s_ and ε_∞_ are limit of the permittivity at low and high frequencies, εo is the permittivity of free space, τ is the relaxation time, α is the distribution parameter describing the symmetrical broadening of the relaxation loss peak, and σi is the ionic conductivity. The relaxation function describes the loss peak and the decrease in ε’ Gulich et al. [1]. If α is set to 0, the model is reduced to Debye model. Both Cole–Cole and Debye model were tested to fit the measured dielectric properties of artificial sweat in both diluted and dehydration states. However, with the given measurements (1–20 GHz), Cole–Cole model was a better fit for the loss peak observed at higher frequencies. The deviation from the Debye model (α ≠ 0) was connected with the correlations of the reorienting dipoles. According to the Jonscher’s relationship, the alpha parameter reflects stronger long-time correlations. 

**Figure 7:**
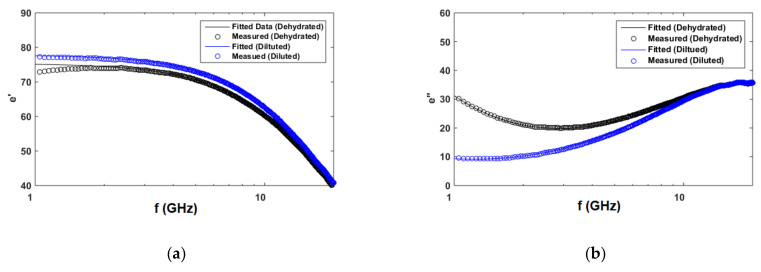
Measured and fitted: (**a**) real part of complex permittivity (ε’). (**b**) Imaginary part of complex permittivity (ε”) for diluted artificial sweat (0.05% NaCl, 0.01% for each urea, lactic acid, and KCl) and dehydrated artificial sweat (0.5% NaCl, 0.1% for each urea, lactic acid, and KCl).

Table 4 shows the calculated Cole–Cole model parameters for artificial sweat representing normal and dehydrated states, while Figure 7 shows the fitted data. ε_∞_ was calculated as 5.1398 using equation in Peyman et al. [2] and La Gioia et al. [27], which was a function of measurement temperature and concentration. 

#### 3.3.4. Variation of Components Concentrations

**Figure 8:**
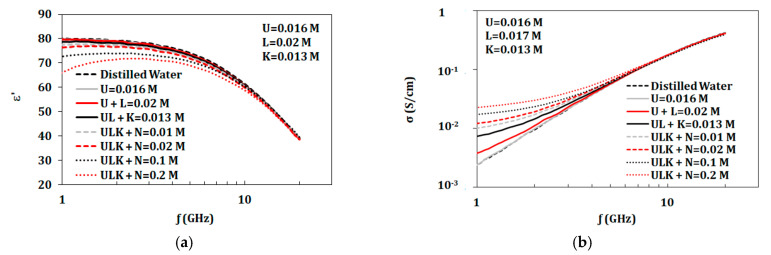
Measured dielectric properties for NaCl solutions at ion concentrations from 0.01 to 0.2 mol/L with a base of 0.013 mol/L KCl, 0.016 mol/L urea, and 0.02 mol/L lactic acid at temperature 23 °C in the range of 1–20 GHz: (**a**) dielectric constant and (**b**) conductivity(“M” refers to mol/L).

**Figure 9:**
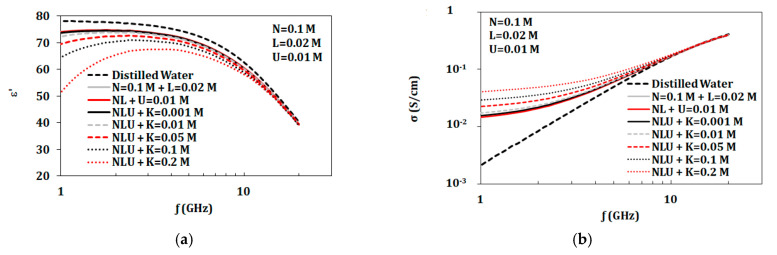
Measured dielectric properties for KCl solutions at ion concentrations from 0.01 to 0.2 mol/L with a base of 0.1 mol/L NaCl, 0.016 mol/L urea, and 0.02 mol/L lactic acid at temperature 23 °C in the range of 1–20 GHz: (**a**) dielectric constant and (**b**) conductivity (“M” refers to mol/L).

The artificial sweat mixture consists of NaCl, KCl, urea, and lactic acid. In this section, the effect of varying one component in the presence of the other three components was studied. The constant components are set at the concentrations appropriate for dehydrated sweat. The variable component is adjusted over the range of 0–0.2 mol/L. This range is chosen as it is common for all tested components. Figure 8 and Figure 9 show the effect of varying NaCl and KCl, respectively. Variations of lactic acid and urea were also tested in the range of 0–0.2 mol/L in the presence of other three components where no changes were observed. 

## 4. Discussion

In this paper, artificial sweat is prepared at concentrations representing different hydration states. Normal (termed diluted) and dehydrated sweat with overall concentrations of 13.1 and 131 mmol/L, respectively, are synthesized and characterized in the range of 1–20 GHz. The measurements in Figure 6 show distinct values of dielectric properties at different hydration states. Changing pH level from 4 to 6.5 does not alter the electrical properties of prepared mixtures. Moreover, measured diluted and dehydrated artificial sweat samples are fitted to a Cole–Cole model with parameters shown in Table 4. The measurements and models are useful tools for designing and testing sweat-based applications. 

**Figure 10:**
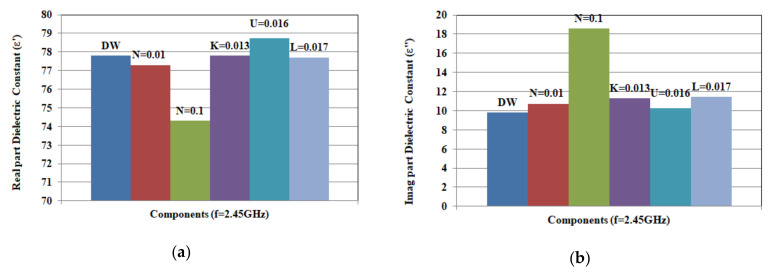
Measured complex permittivity: (**a**) real part (ε’) and (**b**) imaginary part at 2.45 GHz for aqueous solutions of single component (N = 0.01, 0.1 mol, K = 0.013 mol, L = 0.017 mol, and U = 0.016 mol). DW is distilled water.

**Figure 11:**
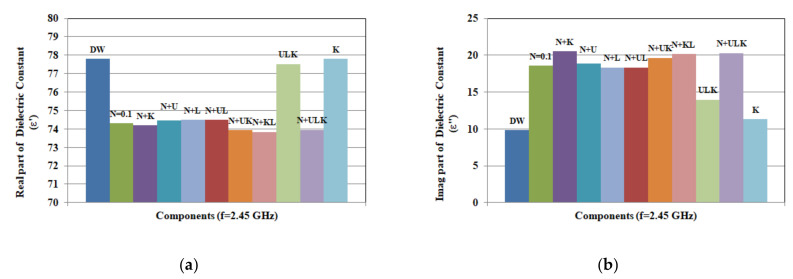
Measured complex permittivity: (**a**) real part (ε’) and (**b**) imaginary part at 2.45 GHz Figure 6. (N = 0.1 mol, K = 0.013 mol, L = 0.017 mol, and U = 0.016 mol).

To explore the influence of components of the solutions, two sets of data are recorded and plotted in Figure 10 and Figure 11. The given concentrations are chosen to represent different hydration states. To study the effect of changing concentration, properties of solutions at different concentrations are compared at 2.45 GHz. This is a frequency of operation for different wireless and sensing applications, as well as wearable devices [28,29,30,31,32,33]. The first set of data in Figure 10 presents the real and imaginary part of the dielectric constant of solutions formed from single components at 2.45 GHz. With these concentrations, Figure 10 shows that NaCl has the greatest effect on changing dielectric constant, followed by urea, KCl, and lactic acid. On the other hand, NaCl has the greatest impact on varying conductivity, while urea has the least compared to distilled water values. The second set of data in Figure 11 shows comparison of dielectric properties (real and imaginary part at 2.45 GHz) for solutions of different mixtures of components with given range of concentrations shown in Table 5.

The given results show that interaction between components forming mixtures could have an effect on determining the properties of the aqueous solutions. Dielectric properties of the mixture of urea, KCl, and lactic acid are comparable to distilled water, however, change remarkably by adding 0.1 mol/L of NaCl. On the other hand, urea, lactic acid, and the combination of lactic acid and urea slightly increase dielectric constant of aqueous solution of 0.1 mol/L NaCl. 

As for conductivity, NaCl solutions and the dual solution of NaCl and KCl have the highest values. 

However, when NaCl is mixed with urea, lactic acid, or combination of both, the mixture of urea, lactic acid, and KCl shows higher conductivity values compared to single component solutions (K, L, and U). However, the conductivity is still lower than 0.1 mol/L solution of NaCl. To validate the given results, solutions of single and combined components are also characterized at 1, 5, and 10 GHz. The recorded data are compared to results at 2.45 GHz. Measurements of permittivity and conductivity at different frequencies show similar variations to those at 2.45 GHz.

**Figure 12:**
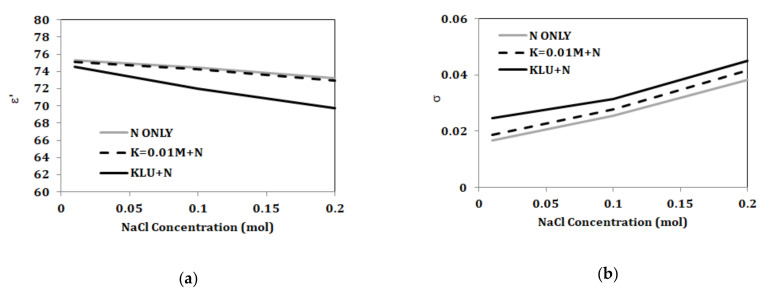
Measured (**a**) real part of complex permittivity (ε’) and (**b**) conductivity versus concentration at 2.45 GHz for aqueous solutions of NaCl, NaCl with K = 0.013 mol/L, NaCl with K = 0.01 mol/L, L = 0.02 mol/L, and U = 0.01 mol/L (“M” refers to mol/L).

**Figure 13:**
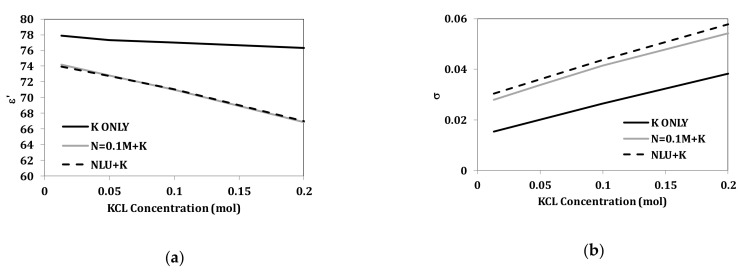
Measured (**a**) real part of complex permittivity (ε’) and (**b**) conductivity versus concentration at 2.45 GHz for aqueous solutions of KCl, KCl with N = 0.1 mol/L, KCl with N = 0.1 mol/L, L = 0.02 mol/L, and U = 0.01 mol/L (“M” refers to mol/L).

Further analyses for changing NaCl and KCl concentrations in the presence of other components are presented in Figure 12 and Figure 13. Figure 12 shows comparison of properties for solely NaCl, NaCl with 0.013 mol/L KCl, and NaCl with 46 mmol/L of combined KCl, urea, and lactic acid. KCl with concentration of 0.013 mol/L does not alter the dielectric constant of NaCl solutions but does increase the conductivity. With 46 mmol/L of combined KCl, urea, and lactic acid, NaCl solutions show changes in properties, such as decrease in dielectric constant and increase in conductivity. Figure 13 shows comparison of properties for solely KCl, KCl with 0.1 mol/L NaCl, and KCl with 118 mmol/L of combined NaCl, urea, and lactic acid. After adding 0.1 mol/L of NaCl, the properties of the KCl solutions change. On the other hand, adding lactic acid and urea with 0.1 mol/L of NaCl show increased conductivity and similar permittivity when compared to mixtures of KCl and 0.1 mol/L NaCl. 

In a large number of wearable devices and biosensors, the sensing decision depends on recording changes in electrical properties of tested solutions and biofluids. Thus, it is crucial to record and analyze the variations of complex permittivity values with frequency. These values and trends would help in designing biosensors with high sensitivity for sweat monitoring applications. Moreover, the above measurements verify that NaCl, a dominant sweat component [10,11,12,13,14,15], has greatest effect on changing dielectric properties for sweat mixtures.

## 5. Conclusions

This paper provides a novel set of measurements to literature indicating solutions of biologically relevant concentrations of NaCl, KCl, urea, lactic acid, and their mixtures in the band of 1–20 GHz, at temperature of 23 °C, with ionic concentrations in the range of 0.01–1.7 mol/L. NaCl, KCl, urea, and lactic acid represent the four major sweat components. Most of the reported literature examines properties of solutions of single components, specifically NaCl or KCl. However, mixtures of the four major sweat components are studied in detail for the first time in literature. The data presented in this study will allow understanding of microwave interaction with biological tissues, designing and testing new sensors, wearable devices, and therapeutic technologies.

Moreover, this paper presents a novel set of data indicating frequency-dependent electrical properties of artificial sweat (representing normal and hydrated states) and the effect of changing pH level on measured electrical properties. Measured dielectric properties of artificial sweat at both states are fitted using Cole–Cole model. The model parameters describing artificial sweat mixtures are also calculated for the first time in literature in this paper. Additional testing using mixtures of artificial sweat components linked changes in dielectric properties of artificial sweat with varying NaCl concentrations in comparison to property changes obtained when concentrations of other sweat electrolytes were varied (e.g., potassium chloride (KCl), urea, and lactic acid). These data are of highly relevance in designing and testing hydration and sweat monitoring devices.

The data presented in paper are not limited to hydration monitoring but may find application in understanding microwave interactions with human tissues and designing and testing of biosensors and consumer products.

## Figures and Tables

**Table 1:** Single component solutions under test (synthesized by dissolving in 1 L of distilled water).

Tested Component	Concentrations of Components (mol/L)				
NaCl (“N”)	0.01	0.1	0.2	0.34	1.7
KCl (“K”)	0.01	0.05	0.1	0.2	
Lactic Acid (“L”)	0.02	0.06	0.12		
Urea (“U”)	0.016	0.05	0.1	0.2	

**Table 2:** Mixtures under test (synthesized by dissolving in 1 L of distilled water).

Base Component	Concentrations of Tested Components (mol/L)					
K = 0.013 mol	N	0.01	0.1	0.34	1	1.7
N = 0.1 mol	K	0.01	0.05	0.1	0.2	
U = 0.016 mol, L = 0.02 mol, K = 0.013 mol	N	0.01	0.02	0.1	0.2	
N = 0.1 mol, L = 0.02 mol, U = 0.016 mol	K	0.01	0.05	0.1	0.2	
N = 0.1 mol, K = 0.013 mol, U = 0.016 mol	L	0.02	0.06	0.12		
N = 0.1 mol, K = 0.013 mol, L = 0.02 mol	U	0.016	0.05	0.1	0.2	

**Table 3:** Electrical properties for distilled water (DW) and artificial sweat solutions at 2.45 GHz.

Tested Solution	ε’	ε”	σ
Distilled water	77.8	9.8	0.012599
Diluted sweat (13 mmol/L)	76.4218	11.2541	0.015295
Dehydrated sweat (131 mmol/L)	73.9557	20.29	0.03443

**Table 4:** Cole–Cole parameters of artificial sweat mixtures obtained by fitting the experimental data collected from 1 to 20 GHz and at 23 °C to a Cole–Cole model.

Tested Solution	Concentration	εs	τ	α	σi
Diluted sweat	13.1 mmol/L	77.8	8.15 ps	0.005	0.31
Dehydrated sweat	131 mmol/L	75.4	8.1 ps	0.015	1.53

**Table 5:** Component solutions under test (synthesized by dissolving in 1 L of distilled water).

Component (N = 0.1 mol, K = 0.013 mol, U = 0.016 mol, and L = 0.017 mol)
Single component solutions: N = 0.01 mol, N = 0.1 mol, K, L, U
Dual component solutions: N + K, N + L, N + K
Three component solutions: N + KL, N + UL, N + KU, UKL
Four component solution: N + UKL
